# The Effect of *Flammulina velutipes* Polysaccharide on Immunization Analyzed by Intestinal Flora and Proteomics

**DOI:** 10.3389/fnut.2022.841230

**Published:** 2022-01-28

**Authors:** Qiongxin Liang, Qingchun Zhao, Xuting Hao, Jinmei Wang, Changyang Ma, Xuefeng Xi, Wenyi Kang

**Affiliations:** ^1^National R&D Center for Edible Fungus Processing Technology, Henan University, Kaifeng, China; ^2^Functional Food Engineering Technology Research Center, Kaifeng, China; ^3^Joint International Research Laboratory of Food & Medicine Resource Function, Kaifeng, China; ^4^College of Physical Education, Henan University, Kaifeng, China

**Keywords:** *Flammulina velutipes*, *Flammulina velutipes* polysaccharide, immunomodulation, proteomics, intestinal flora

## Abstract

Proteomics and intestinal flora were used to determine the mechanism of immune modulatory effects of *Flammulina velutipes* polysaccharide on immunosuppressed mice. The results showed that compared with the model group, *F. velutipes* polysaccharide could increase thymus and spleen indices and improve thymus tissue structure in mice; IL-2 and IL-4 contents were significantly increased and IL-6 and TNF-α contents were significantly decreased; serum acid phosphatase (ACP), lactate dehydrogenase (LDH) and total antioxidant capacity (T-AOC) activities were increased (*P* < 0.05); in the liver, superoxide dismutase (SOD) and catalase (CAT) activities were increased (*P* < 0.001), while malondialdehyde (MDA) content was decreased (*P* < 0.001). Proteomics discovered that *F. velutipes* polysaccharides may exert immune modulatory effects by participating in signaling pathways such as immune diseases, transport and catabolism, phagosomes and influenza A, regulating the immune-related proteins Transferrin receptor protein 1 (TFRC) and Radical S-adenosyl methionine domain-containing protein 2 (RSAD2), etc. Gut microbial studies showed that *F. velutipes* polysaccharides could increase the abundance of intestinal flora and improve the flora structure. Compared to the model group, the content of short-chain fatty acids (SCFAs) and the relative abundance of SCFA-producers *Bacteroides* and *Alloprevotella* were increased in the *F. velutipes* polysaccharide administration group, while *Lachnospiraceae_NK4A136_group* and *f_Lachnospiraceae_Unclassified* decreased in relative abundance. Thus, *F. velutipes* polysaccharide may play an immunomodulatory role by regulating the intestinal environment and improving the balance of flora.

## Introduction

Immunosuppression is a distinctive feature of immune disorders (1), which predispose to the development of tumors, infections, cardiovascular diseases and diabetes, so the improvement of immune modulatory effects has become an urgent problem. Edible mushrooms are collected and/or cultivated worldwide and considered to be an important part of a healthy human diet (2, 3). In recent years, the immune modulatory effect of edible mushrooms polysaccharides has attracted much attention (4). *Schizophyllum commune* polysaccharides can increase RAW264.7 cell activity and promote the production of large amounts of NO by cells to exert immune effects (5). *Hericium erinaceus* polysaccharide (HEP) can improve immune function by enhancing cellular and humoral immunity, macrophage phagocytosis and NK cell activity in mice, in addition, Sheng et al. found that HEP can upregulate intestinal SIgA secretion and activate MAPK and AKT cell signaling pathways (6).

*Flammulina velutipes* is one of the most popular edible mushrooms that contain triterpenes, polysaccharides, sterols and vitamins. *F. velutipes* polysaccharides have anti-inflammatory, antioxidant, anti-aging, immune modulatory, and intestinal flora activities (7–10). Earlier experiments of our group found that *F. velutipes* polysaccharides were non-toxic and could promote intestinal motility and improve constipation in rats (11). Liang obtained two polysaccharides CHFVP-1 (24.44 kDa) and CHFVP-2 (1,497 kDa) from *F. velutipes* and found that CHFVP-1 had procoagulant activity *in vitro* (12). A review of the literature shows that *F. velutipes* polysaccharides can modulate the intestinal microbiota of healthy mice and has potential immunomodulatory abilities (13, 14). However, the target proteins of immunomodulatory effects of this biologically active polysaccharide in immunocompromised mice remains to be investigated.

Proteomics can elucidate the composition of proteins and their action patterns in tissues and cells at a holistic level, can rapidly and precisely identify the key functional proteins between polysaccharides and immunity, lay the foundation for the study of disease mechanisms, drug action targets and new drug development (15–18). Zhao et al. investigated the key proteins and immune-related pathways stimulated by LPS after pretreatment with Hippophae rhamnoides polysaccharide in IPEC-J2 cells by proteomics techniques, and identified 42 key proteins related to immune pathways, and MAPKs/NF-κB signaling pathway may be the target for the efficacy of the drug (19). Yang et al. analyzed the effect of Taishan *Pinus massoniana* pollen polysaccharide on chicken peripheral blood lymphocyte proteome and identified 10 differentially expressed candidate proteins. Candidate proteins, and some differentially expressed proteins were associated with host innate immune response, stress-induced immune response and lipid synthesis-related pathways (20). The intestine is the largest immune organ and contains a large number of immune cells (21). The intestinal flora and its metabolites can play an important role in host immune homeostasis by promoting the development of the immune system, activating the immune response and regulating immune cell function (22). Numerous studies have shown that a variety of plant-derived polysaccharides can act directly as prebiotics or indirectly induce changes in the intestinal flora (23). Chen et al. found that *food* polysaccharide could reduce intestinal damage in immunosuppressed mice, regulate the composition of intestinal microorganisms, increase the levels of SCFAs, and exert intestinal immunomodulatory effects (24). *Coptis chinensis* Franch polysaccharides, when utilized by the intestinal flora, dynamically regulates the diversity, composition and distribution of the intestinal flora and has a regulatory effect on the intestinal immune microenvironment (25). The use of proteomic techniques and flora analysis will help to explore the mechanism of action of polysaccharide immunomodulation. Therefore, the immunosuppression model was established to investigate the immune modulatory mechanism of *F. velutipes* polysaccharides in immunocompromised mice, and to infer the target proteins of *F. velutipes* polysaccharides by proteomics *in vivo*.

## Materials and Methods

### Materials and Reagents

Cyclophosphamide: Jiangsu Hengrui Pharmaceutical Co., Ltd (batch number: 20031125); *Lentinus edodes* Polysaccharide Tablets: Hubei Guangren Pharmaceutical Co., Ltd (batch number: 1909080); Biochemical kit: Nanjing Jiancheng Institute of Biological Engineering; ELISA kit: Beijing Si Zhengbai Biotechnology Co., Ltd; Paraformaldehyde fixative: Wuhan Xavier Biotechnology Co., Ltd; antibody TFRC: abcam; antibody RSAD2: Wuhan Sanying Biotechnology Co. Multiskan GO full wavelength enzyme standardizer: Thermo Fisher; Analytical balance: Sartorius.

### Extraction of Polysaccharides

*Flammulina velutipes* polysaccharides was extracted according to the literature (26) with the extraction rate of 1.49%.

### Animals

Specific pathogen free (SPF) grade, male, Kunming (KM) mice, 4–5 weeks old, 18–22 g. All mice were placed in a light-dark cycle at 24 ± 2°C for 12 h. Mice were acclimated for 7 d before the start of the experiment and were fed standard chow and had free access to water. Animals were acclimatized and fed for 1 week. The study received ethical approval from the Ethics Committee of Henan University School of Medicine (HUSOM2021-76) and was conducted in accordance with the guidelines of the Ethics Committee of the Animal Experimentation Ethics Committee.

### Establishment of Immunosuppressed Mouse Model

Fifty-four mice were randomly divided into 6 groups of 9 mice each according to body weight, namely, blank group (BC), model group (MC), positive control group (PC), high-dose group (HD, 200 mg/kg), medium-dose group (MD, 100 mg/kg) and low-dose group (LD, 50 mg/kg) of *F. velutipes* polysaccharide. The BC and MC groups were given saline (0.1 mL/10 g) daily by gavage, the PC group was administered *Lentinus edodes* polysaccharide tablets by gavage at 3 mg/kg body weight as a positive control once daily for 21 d. Except for the BC group, which was administered saline intraperitoneally, the remaining groups were molded with CTX at 80 mg/kg intraperitoneally on days 18, 19, 20, and 21 of the experiment.

### Measurement of Organ Indices

The mice were fasted for 12 h after the last administration, and the body weight of each group was weighed. After removing the eyeballs, they were executed. The thymus and spleen tissues of the mice were immediately taken, washed with PBS solution, blotted dry on filter paper and weighed. The immune organ indices of mice were calculated according to the following formula.


$$\begin{array}{l} \text{Immune organ index }=\text{ weight of thymus or spleen }\left(\text{mg}\right)/\\ \text{body weight}\left({\text{g}}^{*}10\right) \end{array}$$

### Determination of Lactate Dehydrogenase and Acid Phosphatase Activity

The prepared sera were taken and the activity of LDH and ACP were determined by microplate method and microenzymatic assay, respectively, according to the instructions of the kit.

### Determination of Cytokine Content in Mice Serum

Blood was taken into 1.5 mL EP tubes by the eyeball removal method, and serum was prepared by centrifugation at 4°C and 3,500 r/min for 10 min. The cytokines interleukin 2 (IL-2), interleukin 4 (IL-4), interleukin 6 (IL-6), and tumor necrosis factor α (TNF-α) were detected in the serum by ELISA according to the kit instructions.

### Determination of Antioxidant Stress Capacity

The prepared serum was taken and the total antioxidant capacity (T-AOC) level in mice was determined according to the kit instructions. The mouse liver tissues were weighed accurately, and 10% tissue homogenate was prepared by homogenization method under ice water bath conditions according to the ratio of weight (g) to volume (mL) of 1:9, and the supernatant was centrifuged at 2,500 rpm/min for 10 min. The supernatant was taken and the malondialdehyde (MDA), superoxide dismutase (SOD) and catalase (CAT) were measured in mouse liver tissues according to the kit instructions, respectively.

### Pathological Observation

The mouse thymus specimens were rinsed with PBS, placed in 4% paraformaldehyde solution for fixation, dehydrated with gradient ethanol solution, and paraffin-embedded sections. H&E staining was performed by hematoxylin-eosin for 5 min, followed by dehydration, sealing of the sections with neutral gum, microscopic examination, and image acquisition for analysis.

### Proteomics Studies

The spleens of mice in the BC, MC, and HD groups were taken, and after washing, the spleen tissues of each three mice were combined into one sample, and the obtained samples were subjected to proteomic assay (27).

### Western Blotting Analysis

According to the amount of protein supernatant, 1/4 of loading buffer was added and mixed, and then heated at 100°C for 10 min. Forty microgram of sample protein solution was taken and analyzed by SDS-PAGE method for protein TFRC and RSAD2.

### Diversity Sequencing of Cecum Contents Flora

Under aseptic conditions, the cecum contents of mice were taken, the contents of three mice from each group were pooled, and the obtained samples were sequenced for flora diversity analysis (28).

### Content Determination of SCFAs

The contents of acetic acid, propionic acid and butyric acid in cecum contents were analyzed with reference to literature (29).

### Bioinformatics Analysis

The identified proteins and peptides were firstly obtained under a filtering criterion of 1% FDR (PSM-level FDR ≤ 0.01). The differential proteins were clustered and analyzed by Euclidean distance and systematic clustering method (Hierarchical Cluster). The identified differential proteins were also subjected to GO functional annotation, Pathway enrichment analysis, protein interactions analysis, and subcellular localization analysis.

The forward and reverse reads obtained from double-end sequencing were spliced two-by-two, and after quality filtering to remove chimeric sequences, the final valid sequences obtained were subjected to OTU clustering analysis by Vsearch (1.9.6) (sequence similarity was set to 97%), and the reference database Silva 132 was used for sequence comparison analysis, and the representative sequences of OTU were analyzed for species taxonomy. Based on the analysis results obtained from OTU, sample Alpha diversity analysis, Beta diversity analysis, and colony function prediction were performed.

### Statistical Analysis

The results were expressed after arithmetic mean and standard deviation, and the data were statistically compared for significant differences by one-way analysis of variance (One-Way ANOVA) using SPSS 19.0 software.

## Results

### Effect of *Flammulina velutipes* Polysaccharide on Thymus Index and Spleen Index of Mice

In Table 1, compared with the BC group, the thymus index and spleen index of the MC group was decreased, indicating the model was established. Compared with the MC group, the spleen index of mice in the polysaccharide administration group and the thymus index in the PC and HD groups were significantly increased, the thymus index in the MD and LD groups had no the significant level, but it was an increasing trend compared with the MC group. It showed that *F. velutipes* polysaccharide could improve the atrophy of immunosuppressed mice constructed by CTX, and promote their development and enhance the immunity of the organism.

**Table 1:** Effect of *F. velutipes* polysaccharide on the index of immune organs.

**Group**	Thymus index (mg/10*g)	Spleen index (mg/10*g)
BC	10.96 ± 4.66	26.77 ± 2.14
MC	5.54 ± 1.31^***^	21.28 ± 3.80^*^
PC	9.37 ± 4.06^##^	24.09 ± 3.27
HD	9.63 ± 3.38^##^	30.63 ± 4.53^###^
MD	6.72 ± 0.95	26.91 ± 4.10^#^
LD	6.19 ± 0.99	30.83 ± 8.22^###^

*Compared with BC group:*

*** *P < 0.001*,

* *P < 0.05; Compared with MC group*:

### *P < 0.001*,

## *P < 0.01*,

# *P < 0.05*.

### Effect of *Flammulina velutipes* Polysaccharide on ACP and LDH Activity in Mice

In Table 2, the ACP and LDH activity in serum of MC group was significantly reduced compared with BC group. After the administration treatment, the ACP vitality in the HD group was significantly increased compared with the MC group, and the LDH vitality in both the PC group and the administered group was improved, especially in the polysaccharide administered group. It indicated that the *F. velutipes* polysaccharide is beneficial to improve the ACP and LDH activity in mice.

**Table 2:** Effect of *F. velutipes* polysaccharide on serum ACP and LDH activity.

**Group**	**ACP (unit/100 mL)**	**LDH (U/L)**
BC	7.75 ± 1.26	5703.86 ± 529.04
MC	4.94 ± 1.22^***^	4814.77 ± 706.42^*^
PC	5.27 ± 1.32	5736.72 ± 431.79^#^
HD	6.79 ± 2.00^##^	6761.14 ± 1148.80^###^
MD	5.39 ± 0.80	6376.81 ± 722.82^###^
LD	4.46 ± 0.74	5989.80 ± 1041.74^##^

*Compared with BC group*:

*** *P < 0.001*,

* *P < 0.05; Compared with MC group*:

### *P < 0.001*,

## *P < 0.01*,

# *P < 0.05*.

### Effect of *Flammulina velutipes* Polysaccharide on the Content of Cytokines in Mice Serum

**Figure 1:**
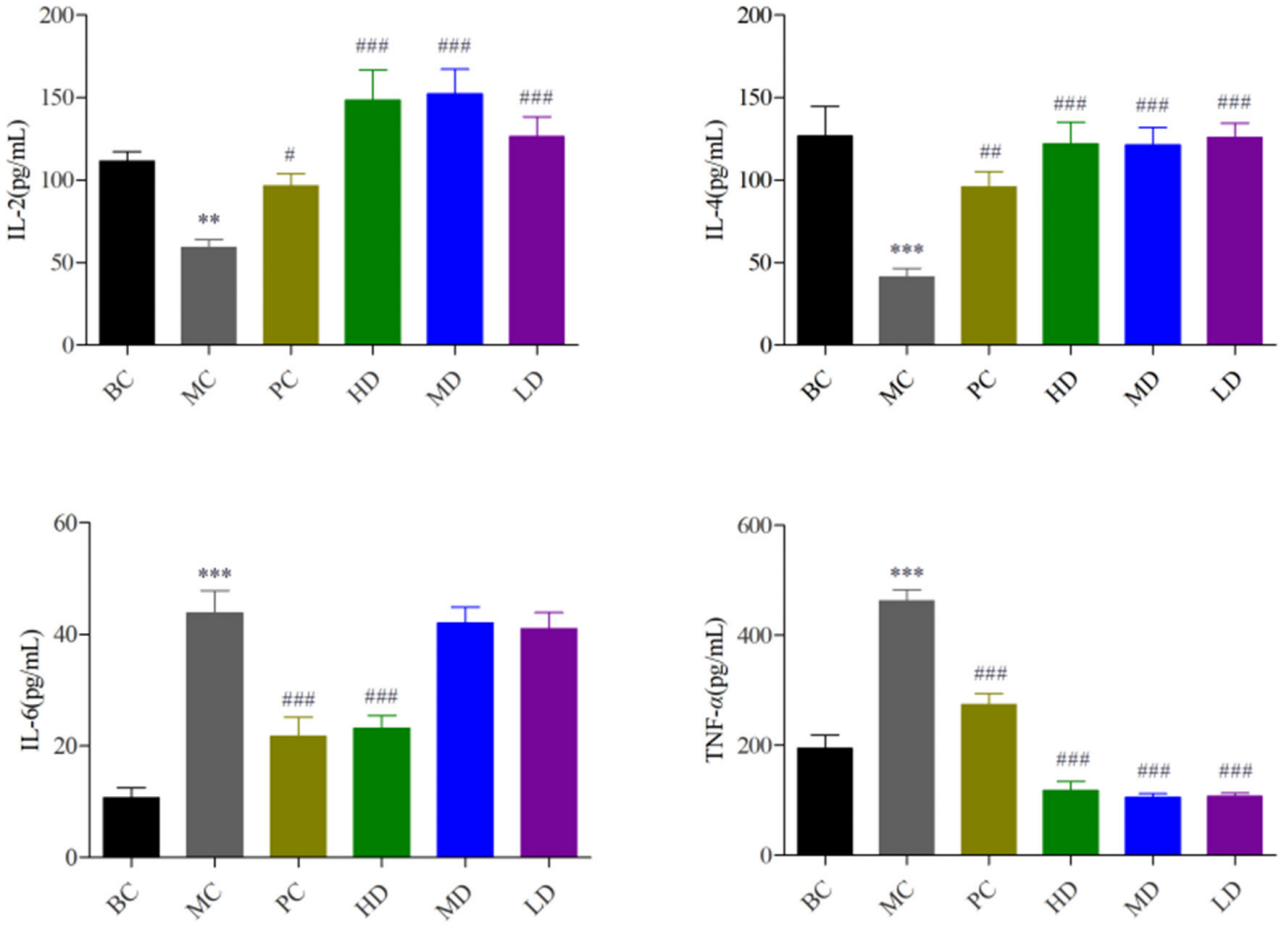
Effect of *F. velutipes* polysaccharide on serum cytokine content. Compared with BC group: ***P < 0.001, **P < 0.01; Compared with MC group: ^###^*P* < 0.001, ^##^P < 0.01, ^#^*P* < 0.05.

In Figure 1, the levels of IL-2 and IL-4 were significantly decreased and the levels of IL-6 and TNF-α were extremely significantly increased in the MC group compared with the BC group (*P* < 0.001), indicating that cyclophosphamide could suppress immune activity in mice. The levels of IL-2, IL-4, IL-6, and TNF-α were improved by administration of the drug, and the best results were achieved in the HD group with highly significant levels (*P* < 0.001). It showed that *F. velutipes* polysaccharide could restore cyclophosphamide-induced immunosuppression and improve immune activity by regulating the levels of cytokines.

### Effect of *Flammulina velutipes* Polysaccharide on Antioxidant Capacity of Mice

**Figure 2:**
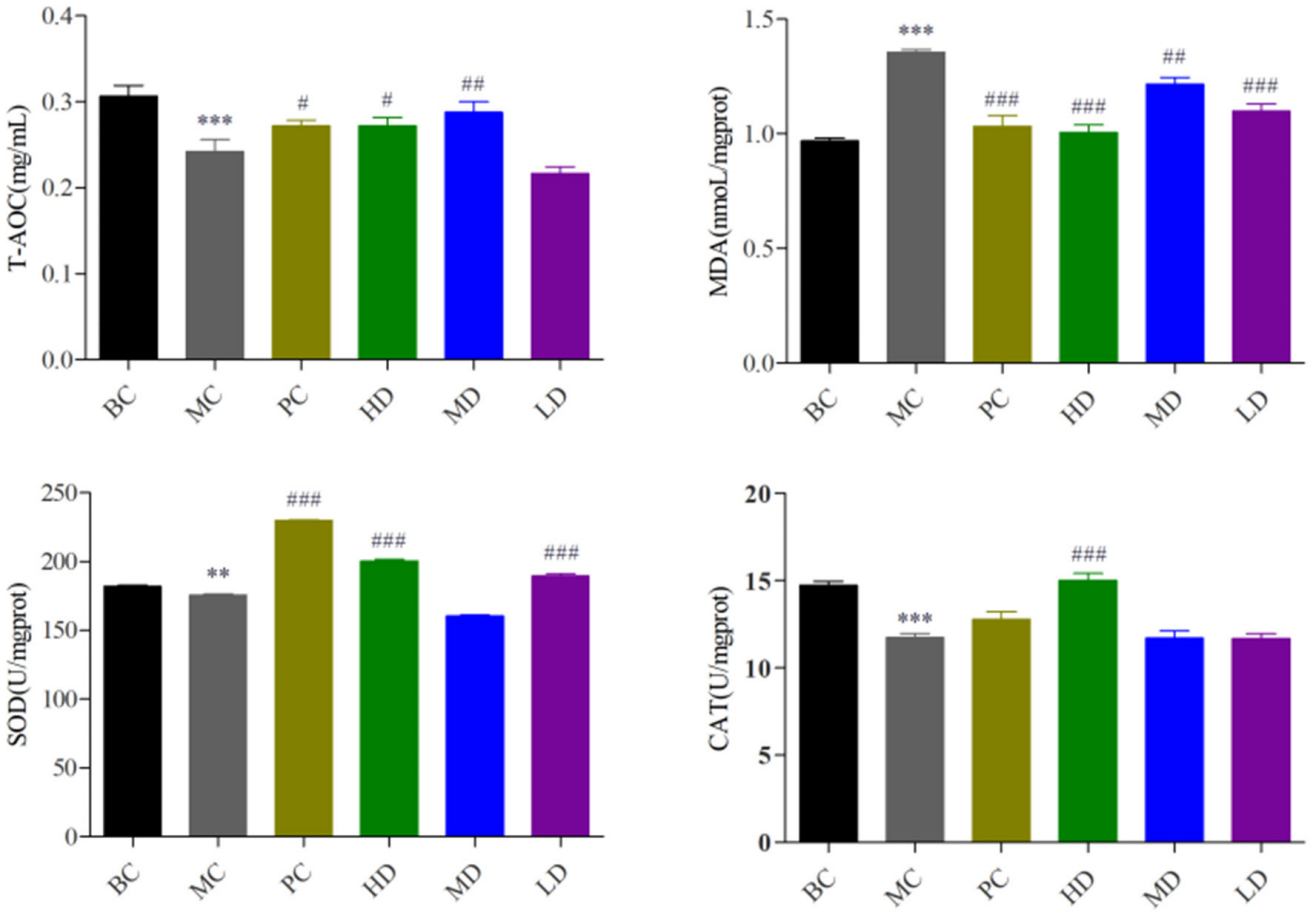
Effect of *F. velutipes* polysaccharide on antioxidant capacity of mice. Compared with BC group: ***P < 0.001, **P < 0.01; Compared with MC group: ^###^*P* < 0.001, ^##^*P* < 0.01, ^#^*P* < 0.05.

In Figure 2, compared with the BC group, the T-AOC content and SOD and CAT activity in the MC group were highly significant decreased (*P* < 0.001) and the MDA activity was significantly increased (*P* < 0.001), indicating the model was established. Compared with the MC group, T-AOC content and SOD and CAT viability values were significantly increased and MDA activity was significantly decreased in the PC group and the high and medium dose administration groups, and SOD viability was significantly increased and MDA activity was highly significantly decreased in the low dose group, and it proved that *F. velutipes* polysaccharide had antioxidant capacity.

### Effect of *Flammulina velutipes* Polysaccharide on Pathological Changes of Mouse Thymus Organs

**Figure 3:**
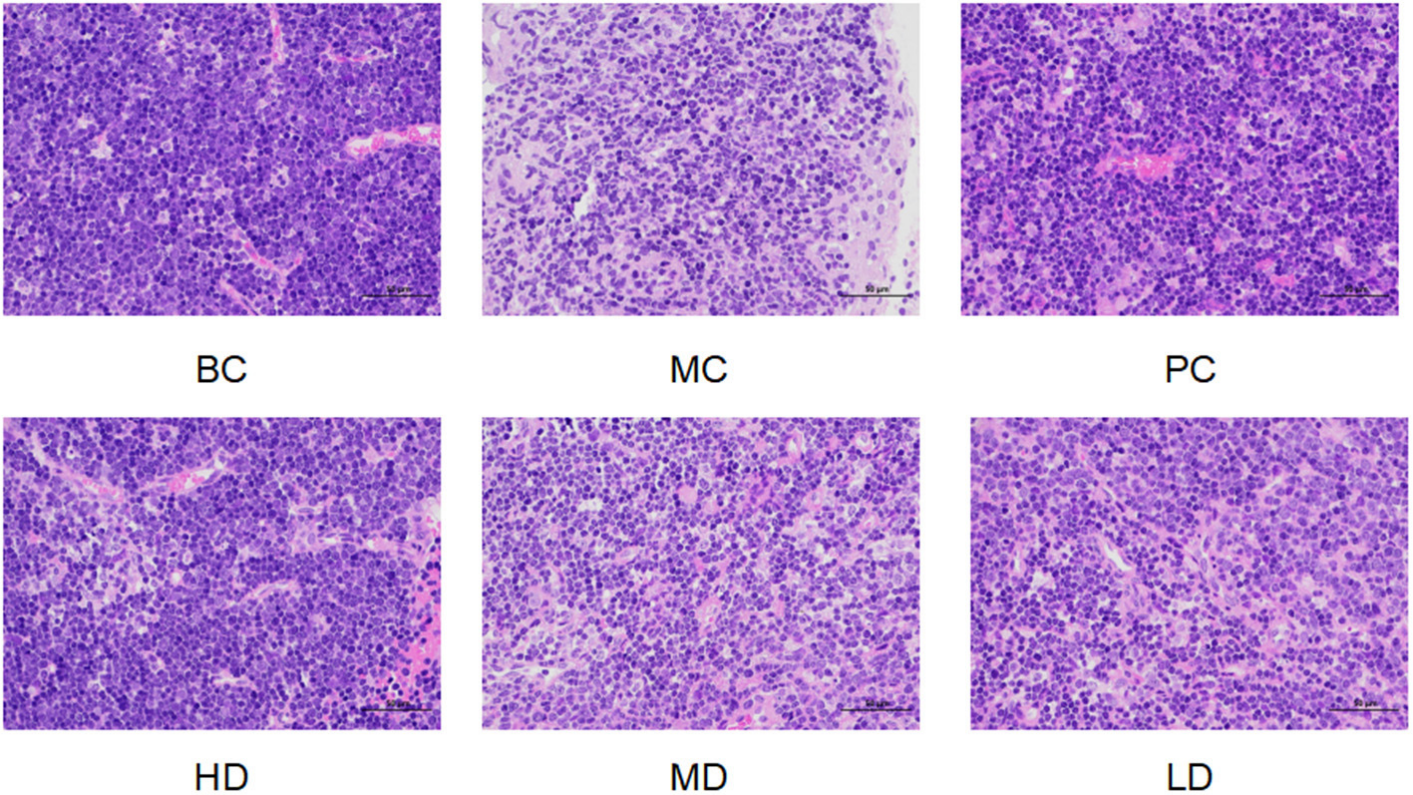
Effect of *F. velutipes* polysaccharide on thymus organ in mice.

In Figure 3, the thymus cells of mice in BC group were abundant and well-arranged, there were no abnormalities, and the thymus staining was darker. Compared with the BC group, the thymus cells in the MC group were arranged in a disorganized and irregular manner with unclear edges, and the decrease of thymus cells led to the overall lighter staining of the thymus. Compared with the MC group, the treatment of *F. velutipes* polysaccharide alleviated the CTX-induced pathological changes in the thymus of mice, indicating that polysaccharide could alleviate the atrophy of the thymus caused by CTX and protect the thymus of mice.

### Protein Identification and Protein GO (Gene Ontology) Annotation Analysis

**Figure 4:**
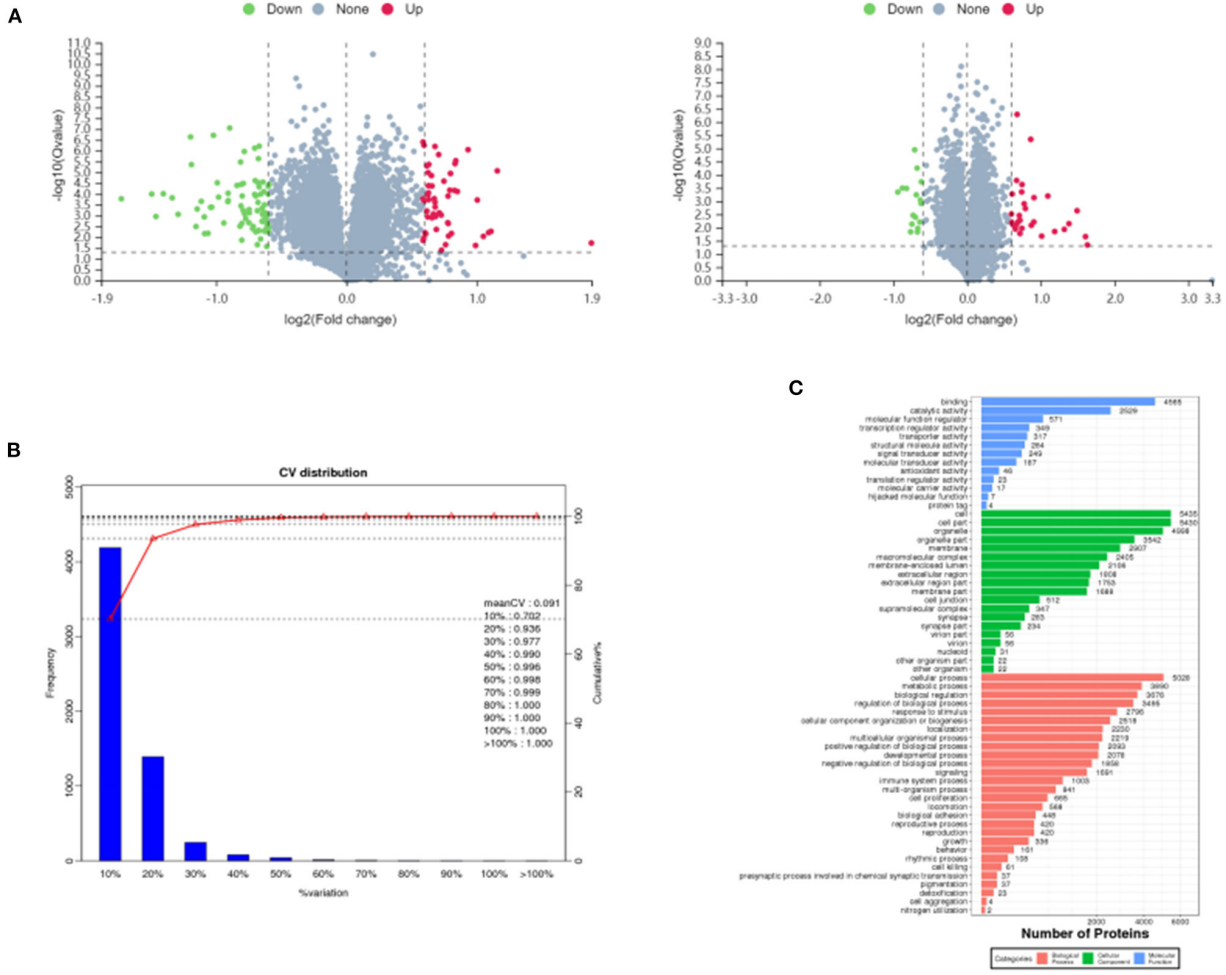
Volcano plots of differentially expressed proteins in MC/BC (left) and HD/MC (right) **(A)**, distribution of CV values of replicate experiments **(B)** and GO annotation histogram **(C)**.

A total of 717,312 secondary spectra were generated for the 3 sets of samples in this experiment. A total of 31,743 peptides and 5,975 proteins were identified under the “1% FDR” filtering criteria. The differentially expressed proteins between groups were compared by screening for differential ploidy and significance. In Figure 4A, the protein quantification results statistics were presented in the form of volcano plots. One hundred and thirty four differentially expressed proteins were identified in the MC group compared with the BC group, of which 52 were up-regulated and 82 were down-regulated. Compared with the MC group, there were 46 differentially expressed proteins after administration of *F. velutipes* polysaccharide treatment, of which 30 were up-regulated and 16 were down-regulated. The reproducibility of the quantification was assessed by the CV value, CV = standard deviation SD/mean, the lower the value, the better the reproducibility. In Figure 4B, the mean CV value was equal to 0.091 and the percentage of proteins with CV values within 20% was 93.6%, and the results indicated that the biological reproducibility was good between sample groups.

All the identified proteins were compared with NR database for GO gene function annotation and enrichment analysis, and it was found that they mainly have molecular functions such as binding, catalytic activity, molecular function regulator, transcription regulator activity, etc.; contain cellular components such as cell, cell part, organelle, membrane, macromolecular complex, etc.; participate in cellular process, metabolic process, biological regulation, regulation of biological process, signaling, immune system process and other biological processes, as shown in Figure 4C.

### GO Enrichment Analysis of Differential Proteins

**Figure 5:**
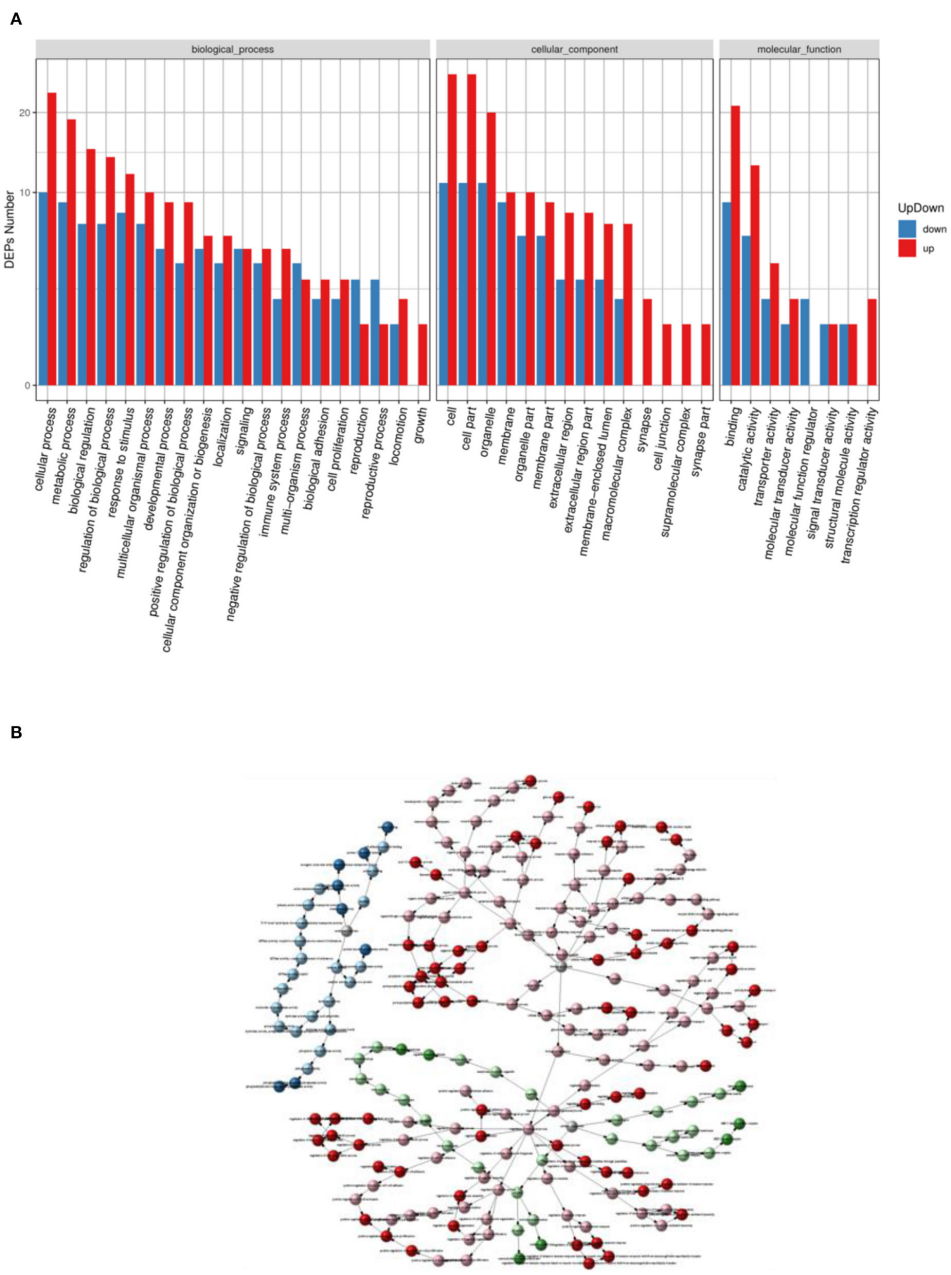
Statistical graph of up- and down-regulation of GO functional classification of differential proteins in HD and MC groups **(A)** and GOterm relationship network graph **(B)**.

The GO entries with significant enrichment of differential proteins were analyzed by clustering in Figure 5A, the horizontal axis represents the GO annotation entries, and the vertical axis represents the up- and down-regulated differential proteins. In Figure 5A, the GO functional classification of differential proteins in the HD group was more up-regulated compared with the MC group, among which cellular process, cell part, cell, binding was the more the GO entries were significantly different. The relationship network was used to observe the relationship between each GOterm in Figure 5B, the significantly enriched GOterms in the HD group and MC group were mainly related to the biological process function.

### Differential Protein Pathway Enrichment Analysis and Subcellular Localization

**Figure 6:**
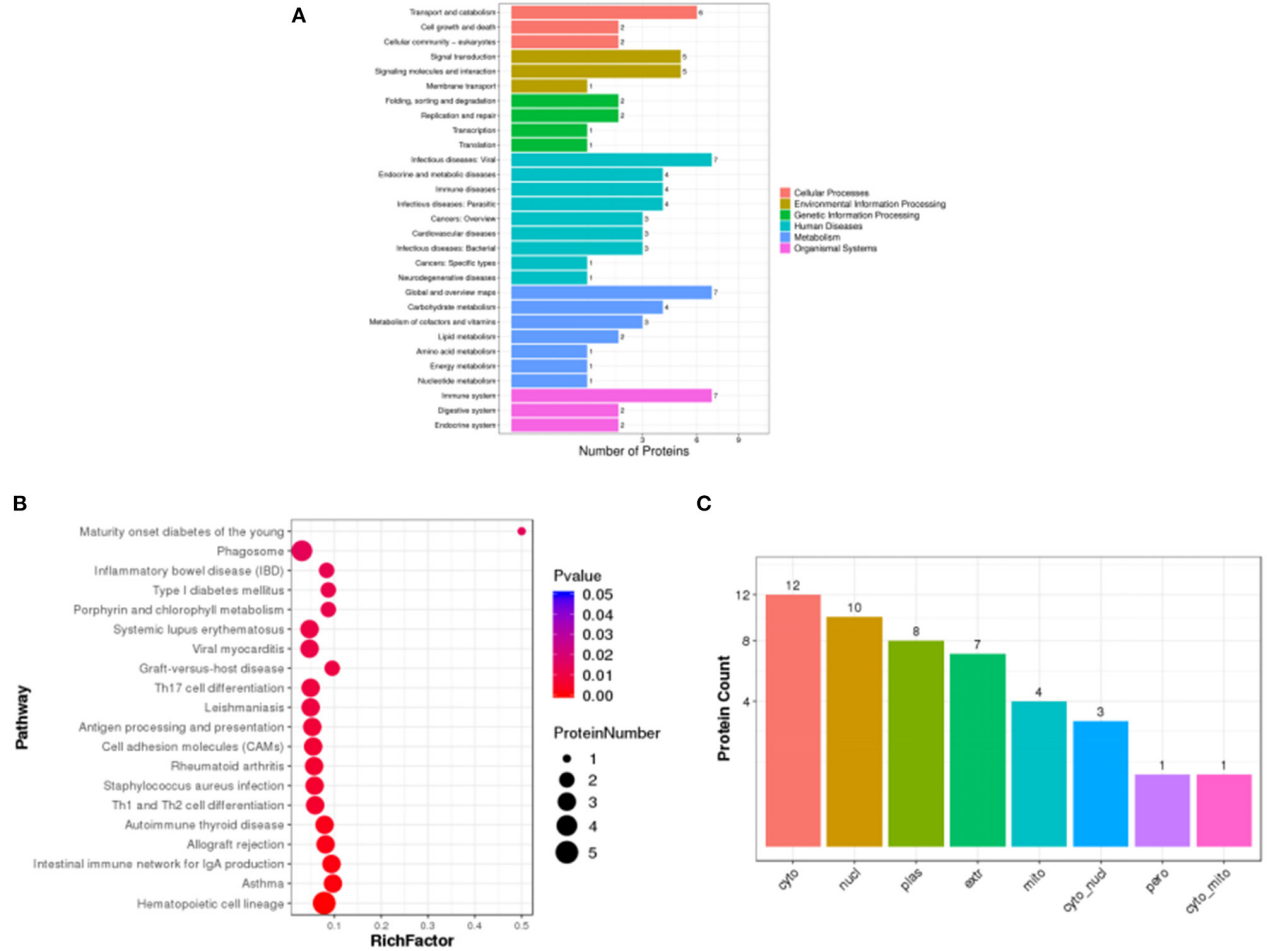
Statistical plots of Pathway classification of differential proteins **(A)**, statistical plots of significantly enriched pathway **(B)** and histogram of subcellular localization **(C)** in HD and MC groups.

Pathway enrichment analysis of differential proteins was performed by the Kyoto Encyclopedia of Genes and Genomes. In Figure 6A, the differentially expressed proteins in the HD and MC groups were mainly involved in transport and catabolism, signal transduction, infectious diseases: viral, immune diseases, carbohydrate metabolism, immune system and other metabolic pathways. The analysis revealed that TFRC (Transferrin receptor protein (1) and RSAD2 (Radical S-adenosyl methionine domain-containing protein (2) protein content changed significantly. The number of differential proteins annotated to the pathway was divided by all the proteins identified to the pathway as the RichFactor, and the larger the value, the larger the proportion of differential proteins in the pathway, and the size of its point represents the number of differential proteins annotated to the pathway. In Figure 6B, differential proteins could play a role in phagosome, antigen processing and presentation, autoimmune thyroid disease, and intestinal immune network for IgA production by participating in the pathway immune modulatory effects.

Subcellular localization of proteins is an important part of protein function annotation. Protein subcellular localization prediction was performed by WoLF PSORT software. In Figure 6C, the differential protein subcellular localization of HD and MC differential proteins were more distributed in cyto (cytosol), nucl (nucleus), plas (plasma membrane) and extr (extracellular).

### Protein Validation

**Figure 7:**
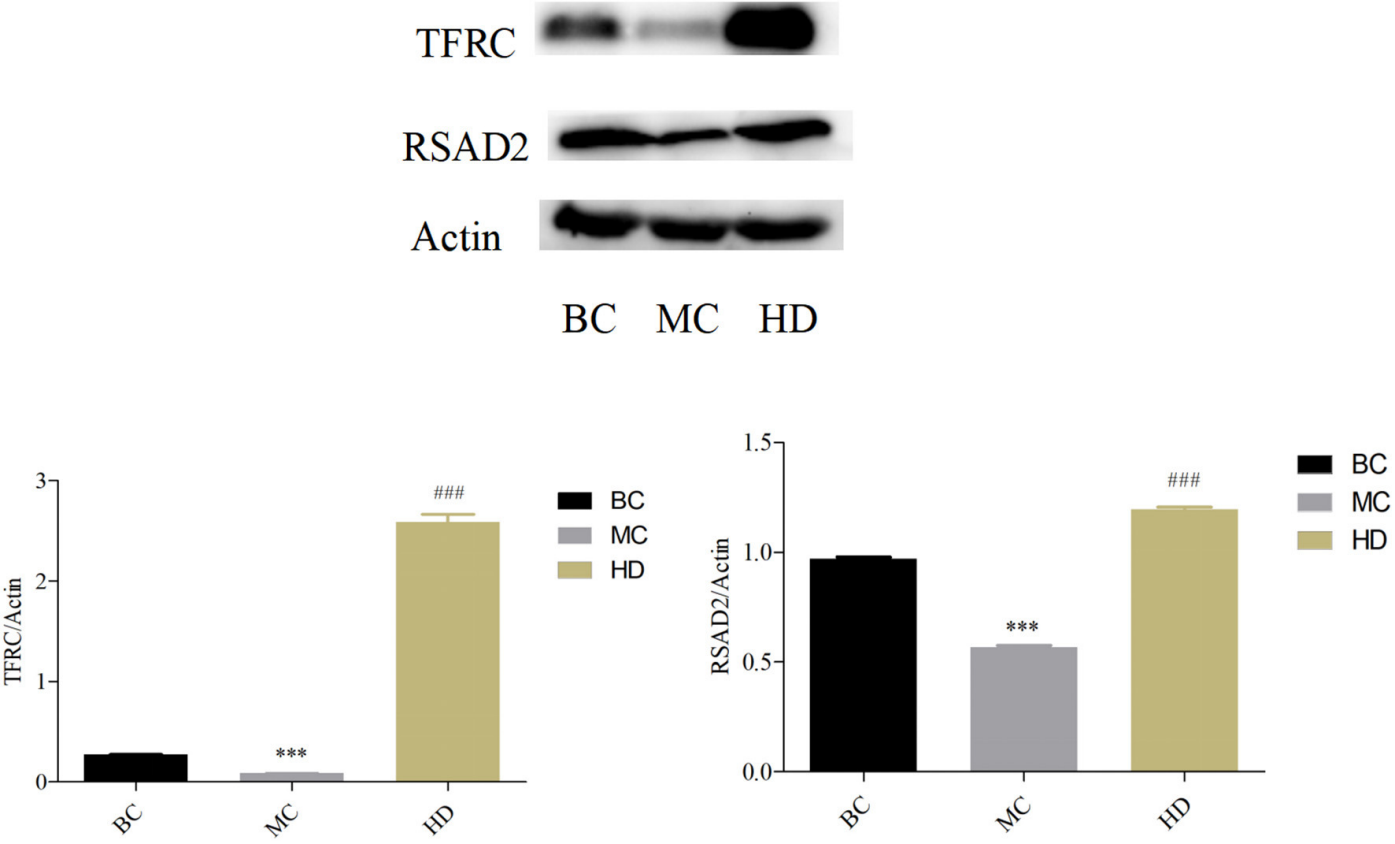
Effect of *F. velutipes* polysaccharide on the expression levels of TFRC and RSAD2 proteins in mouse spleen. Compared with BC group: ***P < 0.001; Compared with MC group: ^###^*P* < 0.001.

In Figure 7, the protein expression in the spleen of TFRC and RSAD2 mice was highly significantly down-regulated in the MC group compared with the BC group (*P* < 0.001), and highly significantly up-regulated in the HD group compared with the MC group (*P* < 0.001), which is consistent with the results of proteomics.

### Taxonomic Analysis of Intestinal Flora Species

**Figure 8:**
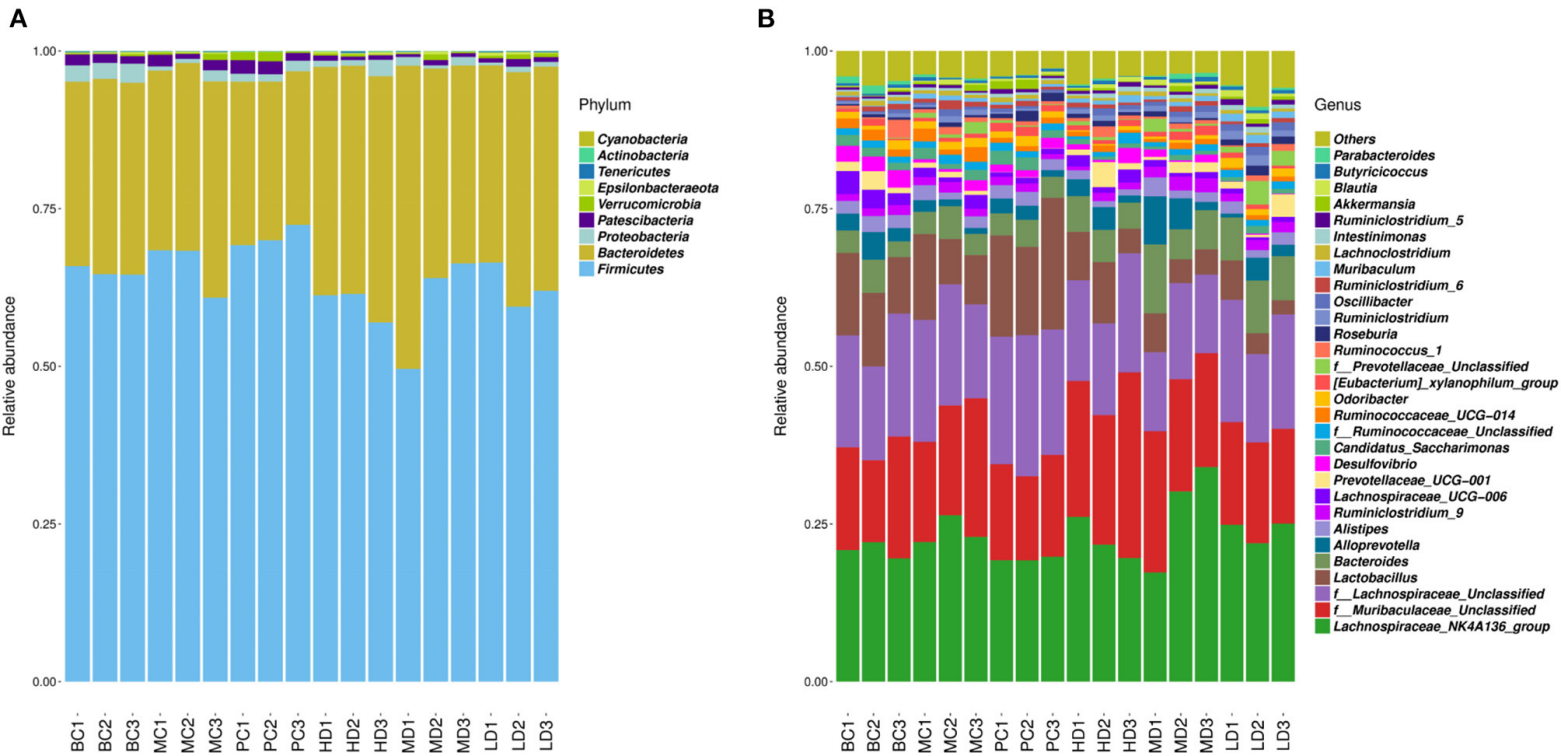
Relative abundance of dominant groups at phylum **(A)** and genus **(B)** level.

OTUs clustering analysis was performed on mouse intestinal microorganisms, and the Top 30 dominant species were selected for analysis by comparing the database for Silva_132 16S rRNA database. In Figure 8A, at the phylum level, the phylum *Firmicutes* and the phylum *Bacteroidetes* were the major dominant groups in the mouse intestinal flora, accounting for more than 95% of all bacteria, followed by the phylum *Proteobacteria*. The relative abundance of *Firmicutes* decreased, the relative abundance of *Bacteroidetes* increased and *Proteobacteria* showed no significant change in the *F. velutipes* polysaccharide administration group compared with the MC group.

To further understand the changes in the intestinal flora, the relative abundance of the dominant flora was analyzed at the genus level in Figure 8B. Compared to the MC group, the relative abundance of *Lachnospiraceae_NK4A136_group* and *f_Lachnospiraceae_Unclassified* was reduced in the blank and high and medium dose administration groups. While the relative abundance of *f_Muribaculaceae_Unclassified, Bacteroides*, and *Alloprevotella* was relatively increased in the HD and MD groups compared with the MC group.

### Colony Diversity Analysis and PICRUSt Functional Prediction Analysis

**Figure 9:**
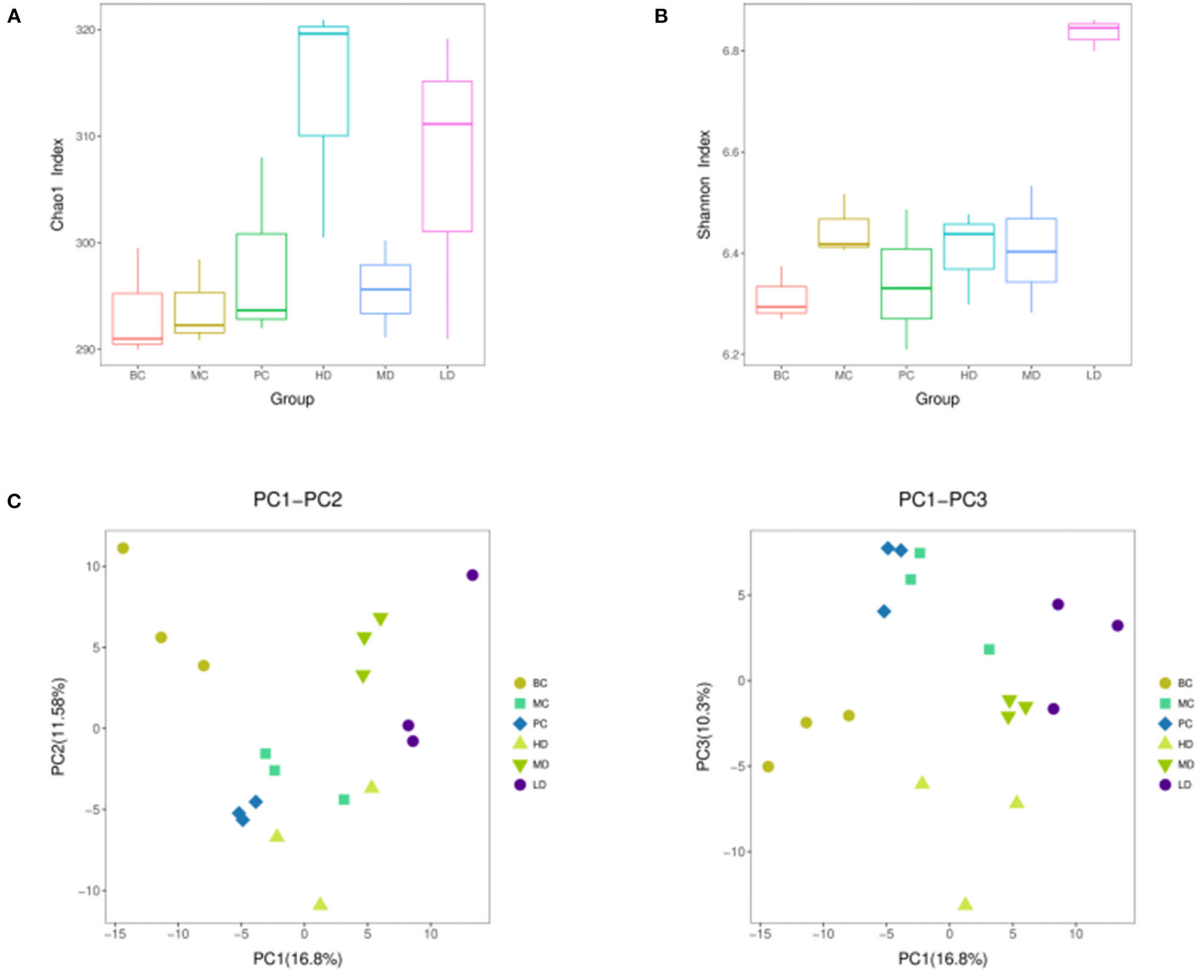
Chao 1 index **(A)**, Shannon index **(B)** based on alpha diversity analysis, PCA analysis performed on the distribution of strain communities **(C)**.

The alpha diversity of the flora was carried out by randomly sampling the sample sequences. Alpha diversity allows assessment of species abundance and diversity. In Figure 9A, the Chao1 index was higher in the HD and LD groups than in the MC group, indicating that the high and low dose groups of *F. velutipes* polysaccharide could increase the abundance of intestinal flora in immunosuppressed mice. Figure 9B showed that the Shannon index value increased in the LD group compared with the MC group, and there was no significant change in the HD and MD groups, indicating that the low-dose administration group could improve the diversity of intestinal flora in mice.

In order to reflect the diversity differences among different samples, the samples were analyzed by beta diversity between groups. In Figure 9C, the composition of intestinal flora of immunosuppressed mice treated with *F. velutipes* polysaccharide was significantly different from that of mice in the MC group, indicating that *F. velutipes* polysaccharide had a good effect on the structural composition of intestinal microorganisms in immunosuppressed mice.

### PICRUSt Functional Prediction Analysis

**Figure 10:**
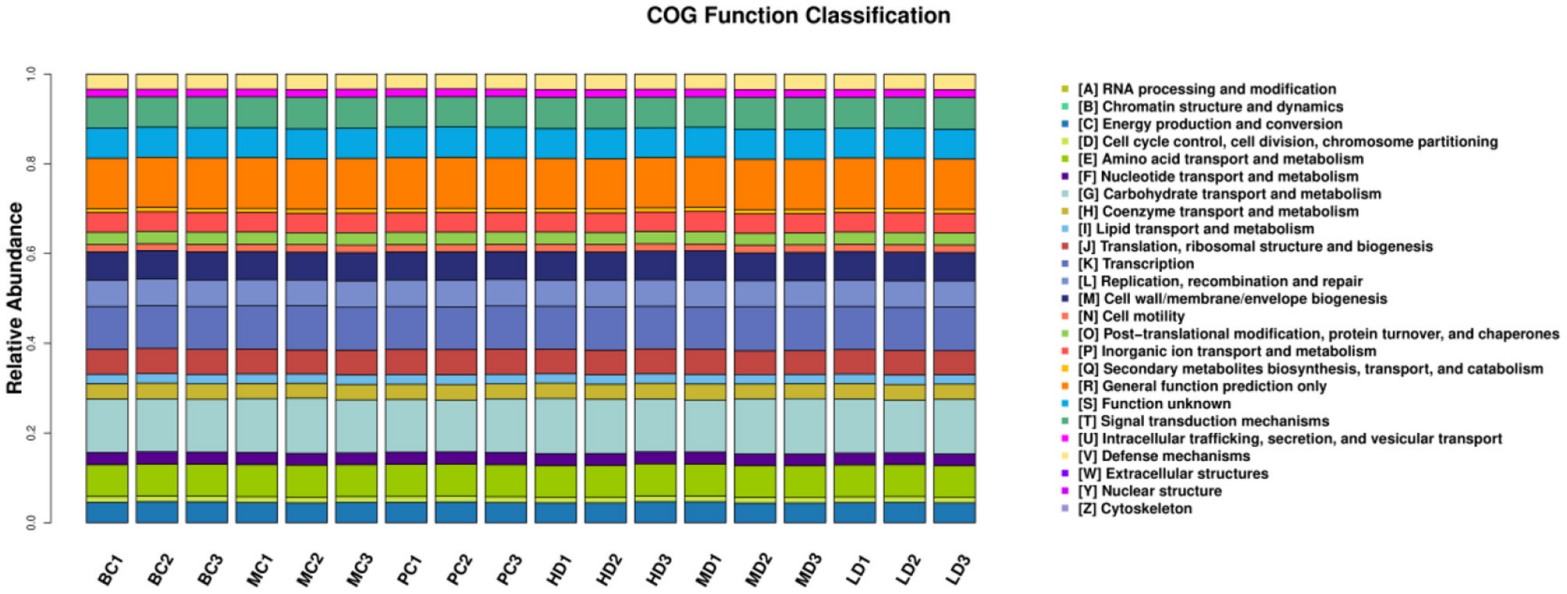
Bar distribution of COG functional abundance.

The metabolic functions of the colony were predicted based on the PICRUSt analysis platform. In Figure 10, the metabolic functions of the colony were concentrated in energy production and conversion, amino acid transport and metabolism, carbohydrate transport and metabolism, lipid transport and metabolism, signal transduction mechanisms, etc. It can be predicted that the intestinal flora may play a regulatory role by affecting the signaling pathways of amino acid, carbohydrate, lipid transport and metabolism and signal transduction.

### Effect of *Flammulina velutipes* Polysaccharide on the Content of SCFAs

The effects of *F. velutipes* polysaccharide on the content of SCFAs were determined by extracting SCFAs from the intestine of mice. In Table 3, compared with BC, the contents of acetic acid, propionic acid and butyric acid were all down-regulated in the MC group, and the contents of acetic acid and butyric acid reached a significant level (*P* < 0.05), indicating that CTX had an inhibitory effect on the production of SCFAs. Compared with the MC group, the content of SCFAs was improved in both the PC and polysaccharide treatment groups, with the content of acetic acid, propionic acid and butyric acid reaching significant levels in the HD group. It was speculated that *F. velutipes* polysaccharide might regulate the changes of intestinal microbial composition by improving the content of SCFAs, with the most obvious improvement in the high-dose group.

**Table 3:** Effect of *F. velutipes* polysaccharide on the content of SCFAs.

	**BC**	**MC**	**PC**	**HD**	**MD**	**LD**
Acetic acid	1.58 ± 0.84	0.35 ± 0.05^*^	1.15 ± 1.11	1.68 ± 1.86^#^	1.14 ± 0.82	0.8 ± 0.55
Propionic acid	3.17 ± 0.47	2.09 ± 0.73	4.47 ± 3.71^#^	5.9 ± 0.77^###^	3.71 ± 0.69	4.11 ± 0.59^#^
Butyric acid	4.54 ± 1.16	2.6 ± 0.87^*^	4.03 ± 2.4	5.32 ± 1.43^##^	3.47 ± 0.69	3.55 ± 1.02

*Compared with BC group*:

* *P < 0.05; Compared with MC group*:

### *P < 0.001*,

## *P < 0.01*,

# *P < 0.05*.

## Discussion

Thymus and spleen are two important immune organs for the proliferation and growth of human immune cells, and the immune organ index is known as the main indicator of immune function (30). It has been shown that polysaccharide can significantly increase thymus index and spleen index in immunosuppressed mice (31, 32). CTX is a chemotherapeutic drug that has a suppressive effect on the body's immune system, which is manifested by a decrease in spleen and thymus index (1). It was found that the thymus and spleen indices of mice in the MC group were significantly lower than those of normal mice, indicating that the immune function of mice was suppressed by CTX, and when the polysaccharide was gavaged, the thymus and spleen indices of mice in each dose group increased to different degrees, and the spleen indices of the polysaccharide administration group and the thymus indices of the HD group increased significantly, and the thymus tissue structure was improved, indicating that *F. velutipes* polysaccharide could improve the immune organ damage and enhance the immunity of the organism, which is consistent with the results of Zhang et al. (33). *Panax japonicus* polysaccharide can improve spleen and thymus indices of immunosuppressed mice induced by cyclophosphamide.

Macrophages arise from the differentiation of monocytes and play a unique role in the immune system with the function of activating the innate immune response (34). Macrophage enzyme activity can reflect the functional status of macrophages. ACP is the marker enzyme of macrophage lysosomal enzymes in higher animals, and its activity reflects the degree of macrophage activation. LDH is an enzyme necessary for intracellular glucose enzymes, and the energy required by phagocytes is also derived from glycolysis. Lactic acid produced during LDH enzymes can cause a decrease in intracellular pH in macrophages, which facilitates the immune response of macrophages and is one of the hallmarks of macrophage activation (35). *Paecilomyces sinensis* polysaccharides can increase the activity of LDH and ACP in rat and human monocytes AMϕ and PMϕ (36). Water-soluble *Ginseng marc* polysaccharide can activate macrophages by regulating the activity of lysosomal phosphatases, affecting the ability of lysosomal enzymes to respond appropriately to exogenous substances and increasing the proportion of phagocytic macrophages (37). The ACP activity in the HD group and LDH in the polysaccharide administration group were significantly increased after the administration treatment, indicating that ACP and LDH activities in immunocompromised mice can be regulated by the *F. velutipes* polysaccharide.

Cytokines have an important regulatory role in cell growth, cell differentiation and cell-cell interactions and have a significant effect on the immune inflammatory response (38). In this experiment, IL-2 and IL-4 levels were significantly decrease and TNF-α and IL-6 levels were significantly increase in the MC group compared to the BC group, indicating that CTX has a suppressive effect on the immune function of the body. IL-2 is mainly expressed by Th1 cells and is a soluble factor that mediates T cell proliferation and has an important role in the innate and adaptive immune system (39). IL-4 is mainly expressed by Th2 cells and is an important anti-inflammatory cytokine that plays an important role in humoral immunity and has an important regulatory role in the immune function and inflammatory process of the body. Wen et al. found that low molecular-weight seleno-aminopolysaccharides significantly increased cyclophosphamide-induced secretion of serum cytokines IL-2 and IL-4 in immunosuppressed mice to restore immune function (40). It is consistent with our research, *F. velutipes* polysaccharides can significantly increase the levels of serum cytokines IL-2 and IL-4 to exert immune modulatory effects. IL-6 and TNF-α have dual effects on the body. Moderate concentrations can enhance the immune function of the organism, regulate a variety of immune cells, and have a protective effect on the organism, but large production and release can disrupt the immune balance of the organism, produce an overly violent immune response, present toxic effects, and cause damage to the organism (41, 42). Cheng et al. (43) showed that *Bupleurum smithii var. Parvifolium* polysaccharides can inhibit lipopolysaccharide induced production of pro-inflammatory cytokines IL-6 and TNF-α. It has been reported that CTX can induce elevation of inflammatory cytokines TNF-α, IL-6 through activation of nuclear factor-κB (NF-κB) and p38 mitogen-activated protein kinase (p38-MAPK) (44–46). *F. velutipes* polysaccharides could exert immune modulatory effects by inhibiting the CTX-induced increase in the levels of pro-inflammatory cytokines IL-6 and TNF-α.

CTX causes immunosuppression along with damage to the liver and antioxidant enzymes, induces oxidative stress in the body, generates large amounts of ROS, causes oxidative damage to immune cells (47–49), and further decreases the immune function of the body. Excess ROS leads to an increase in the membrane lipid peroxidation product MDA (50, 51), which reflects the overall level of lipid peroxidation. SOD is an important oxygen radical scavenger widely present in living organisms, catalyzing the disproportionation of superoxide anions and converting them into hydrogen peroxide, which is decomposed into H_2_O and O_2_ catalyzed by CAT (52–54). Antioxidant the proteins SOD and CAT can act as cellular detoxification systems to prevent ROS damage (55). T-AOC represents the total antioxidant capacity of the organism. It has been shown that polysaccharides can enhance antioxidant activity in immunosuppressed mice (26). Our research found that *F. velutipes* polysaccharide could reduce the level of MDA and alleviate oxidative stress in the body by increasing the activity of SOD, CAT and T-AOC, which is consistent with the results of Xu et al. (56).

Proteomics research involves the large-scale detection, identification and characterization of proteins, making it highly promising for biomarker discovery in many diseases (57). Chen et al. (58) investigated the effect of *Sargassum fusiforme* polysaccharides (SFP) on the antioxidant capacity of liver tissue in mice by proteomics techniques. The effect of SFP on the antioxidant capacity of mouse liver tissues was investigated by proteomic techniques, and 38 out of 49 protein spots were found to be up-regulated and 11 down-regulated. Functional analysis revealed that the differentially expressed proteins were mainly involved in redox, amino acid metabolism and energy metabolism, and the results indicated that SFP could regulate antioxidant enzymes to scavenge excess free radicals and prevent oxidative damage. Jiang et al. investigated the immune modulatory function of *Durio zibethinus* Rind polysaccharide at the proteomic level and found a total of 13 shared differential proteins by comparing the differential proteins in untreated, immunosuppressed and *D. zibethinus* Rind polysaccharide-treated mice. These shared differential proteins were mostly associated with biological functions such as lolalization, biological regulation and immune system process. Liang et al. (59) used proteomics to find that *Nigella sativa* seed polysaccharides could participate in immune regulation by regulating metabolism-related pathways such as Autoimmune thyroid disease, Primary immunodeficiency, and PI3K-Akt signaling pathway through the regulation of differential proteins such as PI3K and PTEN. The aim of this study was to investigate the immune modulatory effects of *F. velutipes* polysaccharide at the proteomic level through the quantitative technique of homogeneous isotope labeling. By comparing the differential proteins in spleen tissues of different treated mice, 52 differential proteins were up-regulated and 82 differential proteins were down-regulated in the MC group compared with the BC group, and 30 differential proteins were up-regulated and 16 differential proteins were down-regulated in *F. velutipes* polysaccharide treated group compared with the MC group. These differential proteins were mostly associated with functions such as biological regulation, immune system process and signal transducer activity, and were involved in transport and catabolism, infectious diseases: viral, immune diseases, phagosome and other pathways to play immune regulatory roles. Differential protein function enrichment analysis revealed that among the immune-related proteins, TFRC and RSAD2 were significantly upregulated in HD compared with MC group. Iron is essential for the generation of immune responses and is required for the growth, proliferation and differentiation of immune cells (60). Iron deficiency leads to a decrease in the number of T cells, a lower proportion of mature T cells and suppressed cytokine synthesis (61–63). Cellular iron uptake is largely dependent on iron transporters (64, 65). The transferrin receptor is a cell surface receptor that mediates iron uptake through receptor-mediated endocytosis and is required for cellular iron uptake (66, 67). It has been suggested that mutations in the transferrin receptor gene may lead to severe combined immunodeficiency (68). Promoting the expression of transferrin receptor protein (TFRC) increases cellular iron uptake and helps to enhance host antitumor immunity (69). RSAD2 is a key enzyme of the innate immune response, localized to the cytoplasmic face of the endoplasmic reticulum (70) and/or to lipid droplets via the N-terminal hydrophobic structural domain (71), and its expression is induced by interferon-dependent or non-dependent pathways (72). The inhibition of GAPDH activity by the ddhCTP product of RSAD2 radical SAM activity may improve the rate of NADPH regeneration by affecting upstream metabolic pathways and increasing the flux of the pentose phosphate pathway (PPP). It is also able to increase the rate of reduction of glutathione disulfide (GSSG) to reduced glutathione (GSH), thus protecting cells from reactive oxygen species (ROS) damage (73). In macrophages, the cellular activity of RSAD2 may provide a protective mechanism for cells against viral infection or other conditions that increase ROS levels (74). The results of the present study showed that *F. velutipes* polysaccharide may alleviate CTX-induced oxidative stress capacity as well as increase the body's iron uptake and immune response through upregulation of TFRC and RSAD2 protein expression, and exert immune modulatory effects through drug metabolism-related pathways such as immune diseases, transport and catabolism, phagosomes and influenza A.

The intestinal flora has an important role in immune system development and regulation of immune function (75). Dysfunctional gut microbial structure affects physiological processes such as energy metabolism, immune regulation and liver injury in humans (76–78). Niu et al. evaluated the effect of *Pinus massoniana* pollen polysaccharides (PPPS) on the intestinal flora of mice by 16S rRNA high-throughput sequencing technology and showed that PPPS can regulate the composition of mouse intestinal microorganisms and increase the proportion of probiotic bacteria, and also regulate the systemic immune system by modulating the immunosuppressive status of lymphocytes in Peyer's patches (79). *N. sativa* seed polysaccharides can exert immune modulatory effects by improving the structure of the intestinal flora, increasing flora diversity, and regulating metabolic pathways such as lipid metabolism, polysaccharide synthesis and signal transduction (59). The results of the present study showed that *F. velutipes* polysaccharides improved the composition and diversity of the intestinal flora of mice in a CTX-induced immunosuppression model. At the phylum level, the thick-walled phylum *Firmicutes* and *Bacteroidetes* were the predominant intestinal flora in mice, it is consistent with the Tremaroli et al. research. *Bacteroidetes* and *Firmicutes* accounted for more than 90% of the total intestinal microorganisms (80). It has been shown that *Bacteroidetes* can interact with cellular receptors using lipopolysaccharide and flagellin components to enhance the immune response through cytokine synthesis (81), and that an increase in thick-walled *Bacteroidetes* promotes energy absorption by the body, leading to obesity (82). In the present study, *F. velutipes* polysaccharides decreased the relative abundance of *Firmicutes* and increased the relative abundance of *Bacteroidetes*, which had a beneficial regulatory effect on the intestinal flora. The literature reports that intestinal microorganisms have a large system of carbohydrate-active enzymes that can further utilize polysaccharides (83). After entering the intestine, polysaccharides are converted into SCFAs by microbial metabolism (84). Short-chain fatty acids are not only an important source of energy for intestinal epithelial cells, but it also regulates the production of inflammatory factors and reduces intestinal inflammation and tumorigenesis (85, 86). Among the various microbial metabolites, acetic acid, butyric acid, and propionic acid are the key bacterial metabolites that promote the development and maintenance of the immune system (87). In the present study, it was found that *F. velutipes* polysaccharides increased the content of acetic acid, propionic acid, and butyric acid, with the most significant increase in the high-dose administration group. Tan et al. (88) found that the intervention of *Bacteroides* reduced the destruction of intestinal flora by LPS treatment, maintained the integrity of the intestinal epithelium, had a role in promoting intestinal homeostasis, and its content was proportional to the acetic acid content in short-chain fatty acids (89). Propionate is a health-promoting microbial fermentation metabolite in the human gut that provides energy to the intestine and plays an inhibitory role in the development of disease (90). Butyrate is the preferred source of energy for colon cells and is locally consumed, and has been more extensively studied in inflammation and cancer, where it inhibits colorectal cancer and inflammation (87). *Alloprevotella* belongs to the genus *Bacteroides* of the phylum *Synechococcus* and is SCFA-producers, whose abundance is negatively correlated with obesity and diabetes (91, 92). *Lachnospiraceae_NK4A136 group* is a discriminatory feature of intestinal dysfunction (93), and Wang et al. (94) studied found that *Chrysanthemum morifolium* polysaccharides could improve intestinal dysfunction by decreasing the abundance of *Lachnospiraceae_NK4A136_group* flora and increasing the relative abundance of beneficial bacteria. In the present study, the relative abundance of *Lachnospiraceae_NK4A136_group* flora was decreased and the relative abundance of *Bacteroides* and *Alloprevotella* was increased in the group administered with high and medium doses of *F. velutipes* polysaccharide compared with the MC group, which regulated the balance of flora in the intestine of mice.

In conclusion, *F. velutipes* polysaccharide can protect immune organs of immunosuppressed mice, improve serum cytokine levels, enhance the antioxidant capacity of the body, promote the increase of intestinal SCFAs content, regulate the expression of proteins TFRC and RSAD2, promote energy metabolism, increase the abundance of flora, improve the structure of flora and maintain the homeostasis of the intestinal environment. The results suggest that *F. velutipes* polysaccharide has potential immune modulatory effects.

## Data Availability Statement

The original contributions presented in the study are included in the article/supplementary material, further inquiries can be directed to the corresponding author/s.

## Ethics Statement

The animal study was reviewed and approved by Ethics Committee of Henan University School of Medicine (HUSOM2021-76).

## Author Contributions

QL and QZ: conceptualization, investigation, methodology, software, and writing-original draft preparation. XH and JW: data curation, formal analysis, and visualization. CM and XX: supervision, software, and validation. WK: resources, funding acquisition, project administration, and writing-reviewing and editing. All authors contributed to the article and approved the submitted version.

## Funding

This work was supported by Major Public Welfare Projects in Henan Province (201300110200), Research on Precision Nutrition and Health Food, Department of Science and Technology of Henan Province (CXJD2021006), and The Key Project in Science and Technology Agency of Henan Province (212102110019 and 202102110283).

## Conflict of Interest

The authors declare that the research was conducted in the absence of any commercial or financial relationships that could be construed as a potential conflict of interest.

## Publisher's Note

All claims expressed in this article are solely those of the authors and do not necessarily represent those of their affiliated organizations, or those of the publisher, the editors and the reviewers. Any product that may be evaluated in this article, or claim that may be made by its manufacturer, is not guaranteed or endorsed by the publisher.
